# The *Arabidopsis thaliana* Kinesin-5 AtKRP125b Is a Processive, Microtubule-Sliding Motor Protein with Putative Plant-Specific Functions

**DOI:** 10.3390/ijms222111361

**Published:** 2021-10-21

**Authors:** Tobias Strauß, Saskia Schattner, Stefan Hoth, Wilhelm J. Walter

**Affiliations:** 1 Institute of Plant Science and Microbiology, Universität Hamburg, 20146 Hamburg, Germany; strauss.1988@web.de (T.S.); saskia.schattner@uni-hamburg.de (S.S.); 2 Medical Department, HMU Health and Medical University, 14471 Potsdam, Germany

**Keywords:** kinesin, *Arabidopsis thaliana*, motor protein, intracellular transport, single-molecule

## Abstract

The formation and maintenance of the mitotic spindle during cell division requires several microtubule-interacting motor proteins. Members of the kinesin-5 family play an essential role in the bipolar organization of the spindle. These highly conserved, homotetrameric proteins cross-link anti-parallel microtubules and slide them apart to elongate the spindle during the equal separation of chromosomes. Whereas vertebrate kinesin-5 proteins are well studied, knowledge about the biochemical properties and the function of plant kinesin-5 proteins is still limited. Here, we characterized the properties of AtKRP125b, one of four kinesin-5 proteins in *Arabidopsis thaliana*. In in vitro motility assays, AtKRP125b displayed the archetypal characteristics of a kinesin-5 protein, a low velocity of about 20 nm·s^−1^, and a plus end-directed, processive movement. Moreover, AtKRP125b was able to cross-link microtubules and to slide them apart, as required for developing and maintaining the mitotic spindle. In line with such a function, GFP-AtKRP125b fusion proteins were predominantly detected in the nucleus when expressed in *Arabidopsis thaliana* leaf protoplasts or *Nicotiana benthamiana* epidermis cells and analyzed by confocal microscopy. However, we also detected GFP signals in the cytoplasm, suggesting additional functions. By generating and analyzing *AtKRP125b* promoter-reporter lines, we showed that the *AtKRP125b* promoter was active in the vascular tissue of roots, lateral roots, cotyledons, and true leaves. Remarkably, we could not detect promoter activity in meristematic tissues. Taken together, our biochemical data support a role of AtKRP125b in mitosis, but it may also have additional functions outside the nucleus and during interphase.

## 1. Introduction

Transport processes and restructuring are an essential part of the cycle of a living cell and are largely mediated by motor proteins in almost all eukaryotic organisms. Kinesins move along microtubules and are, therefore, often involved in processes such as organelle transport, mitotic spindle assembly and maintenance, and chromosome movement. Microtubules serve as a transport structure for the navigation of kinesins. However, the dynamics of microtubules themselves can also be influenced by some kinesins by either stabilizing or depolymerizing them in a controlled manner [1,2]. The highly conserved motor domain forms the catalytic center of these proteins and is capable of hydrolyzing ATP. The ATP-turnover allows a precisely timed binding and unbinding of the motor heads to the microtubule resulting in force transmission [3,4].

All kinesins are classified into 14 families according to their structure. Classification is often, but not always, accompanied by functional clustering. The high variability in kinesin function is due to characteristics outside the motor domain of kinesins. Whereas the variability of structure and function within a kinesin family can be quite high, the kinesin-5 family is highly conserved. The classical role of kinesin-5 in animal systems is the assembly and maintenance of the mitotic spindle during anaphase and metaphase [5,6,7]. Kinesin-5, unlike most kinesins, forms dumbbell-shaped bipolar homotetramers rather than homodimers [8]. It has been shown for Eg5, an animal kinesin-5, that both ends of these tetramers, occupied by motor domains, are able to bind to two microtubules simultaneously and thus cross-link them. Movement of Eg5 along the cross-linked microtubules causes anti-parallel microtubules to be displaced relative to each other. Within the spindle, Eg5 cross-links and displaces the spindle midzone microtubules, ensuring their stability and elongation [9]. Knockout of Eg5 leads to nonpolar spindles and thus problems with the correct division of chromosomes during cell division. This highlights the importance of kinesin-5 in cell division. In contrast to the spindle in animal cells, the plant spindle lacks the centrioles, so their structure differs significantly. Nevertheless, there is evidence that kinesin-5 proteins are also involved and play an important role in the plant spindle: the kinesin-5 AtKRP125c occupied plant spindle microtubules, and a lack of AtKRP125c led to compromised spindle structure and problems with cytokinesis [10,11]. Beyond this, the function of kinesin-5 in plants has been poorly understood, especially with regard to the biochemical and biophysical properties of plant kinesin-5 proteins. Here, we performed in vitro motility assays with a recombinant kinesin-5 from *Arabidopsis thaliana* (AtKRP125b). In the process, we found that AtKRP125b was capable of cross-linking and counter-shifting microtubules in vitro and thus indeed possessed all the properties to fulfill the canonical role in the spindle.

Interestingly, *Arabidopsis* has a high number of kinesin-5 genes compared to most eukaryotic organisms. Whereas most eukaryotes contain only one gene encoding a kinesin-5, Arabidopsis was found to have 4 kinesin-5 genes, namely *AtKRP125a*, *AtKRP125b*, *AtKRP125c*, and *AtF16L2.60* [12,13]. In general, the number of kinesin genes in plants is significantly higher than in other eukaryotes [12]. Most of these additional genes have arisen through gene duplications within specific kinesin families, such as kinesin-7, kinesin-12 or kinesin-14. Gene duplication is often accompanied by the acquisition of plant-specific functions. For example, a large proportion of kinesin-14 takes on a role as a minus-end directed kinesin, replacing dynein, which is absent in plants [14]. Kinesins of the kinesin-7 family are involved in plant-specific processes such as phragmoplast expansion [15]. A role in mitochondria is also speculated [16]. Kinesin-12 motor proteins are also involved in phragmoplast and preprophase band organization, both functions outside their classical role in animal cells [17,18,19]. Therefore, we wondered whether the high number of kinesin-5 genes in *Arabidopsis* may also indicate that kinesin-5 has evolved specific functions in plants and investigated putative cellular functions of AtKRP125b. For this purpose, we examined the *AtKRP125b* promoter activity in transgenic promoter-*GUS* Arabidopsis lines and the intracellular localization of GFP-AtKRP125b after expression in transformed *Arabidopsis* and *Nicotiana benthamiana* cells. The *AtKRP125b* promoter was active in vascular but not in meristematic tissue, whereas the AtKRP125b protein was localized in both the nucleus and the cytoplasm. Our results may indicate that AtKRP125b does not have an exclusive role in the mitotic spindle but may also perform additional functions in the cell during interphase.

## 2. Results

### 2.1. The Kinesin AtKRP125b Moves along Microtubules at a Slow Velocity

**Figure 1:**
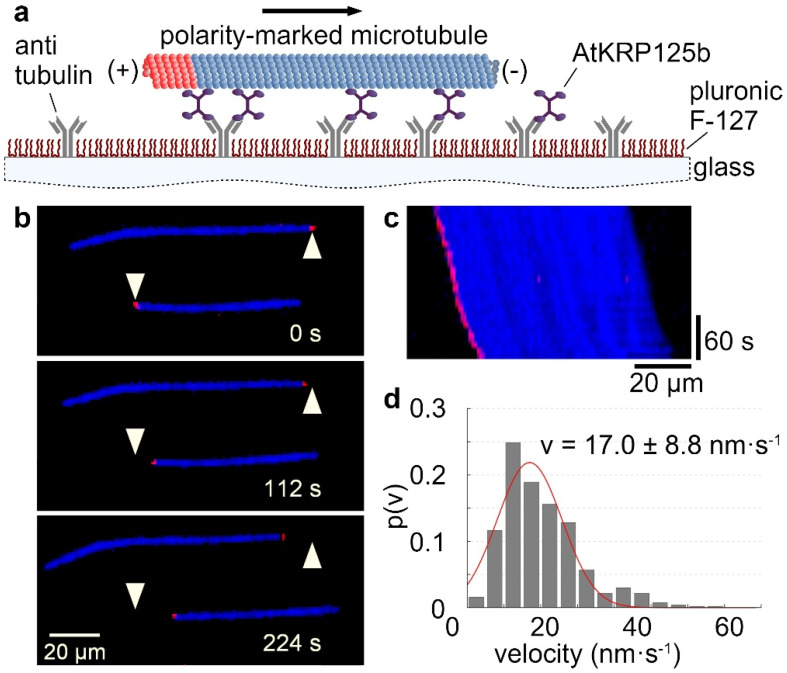
AtKRP125b moves with a slow velocity in in vitro gliding assays. (**a**) Schematic representation of a gliding motility assay with His-tagged full-length AtKRP125b. (**b**) Fluorescent micrographs of polarity-marked microtubules gliding along a AtKRP125b coated surface at different timepoints. The plus end of the polarity-marked microtubule is labelled in red, the remainder of the microtubule is labelled in blue. The starting position of each microtubule is marked by a white arrowhead. (**c**) Kymograph of the lower microtubule shown in (**b**). This space–time plot displays intensity values along the microtubule’s path over time. The velocity can be deduced from the kymograph’s slope. (**d**) Histogram of the mean transport velocities for 504 gliding microtubule filaments. A Gaussian fit (red line) shows a velocity of 17.0 ± 8.8 nm s^−1^ (mean ± SD).

The velocity at which a kinesin moves along microtubules can be determined by using a gliding assay. For this purpose, the recombinant full-length AtKRP125b was bound to a glass surface with antibodies. In an ATP-rich environment, fluorescently labelled microtubules were able to bind to the motor domains of AtKRP125b and were transported by them along the glass surface (Figure 1a–c, Supplementary Movie S1). Analysis of the velocity of microtubule displacement revealed that AtKRP125b displaces microtubules at 17 ± 8.8 nm/s (Figure 1c,d). This velocity is generally quite low compared to other kinesins. However, our observations were comparable to studies on Eg5, which showed that Eg5 moves at a velocity of ~20 nm s^−1^ towards the microtubules plus end [5].

### 2.2. AtKRP125b Is a Processive Kinesin

**Figure 2:**
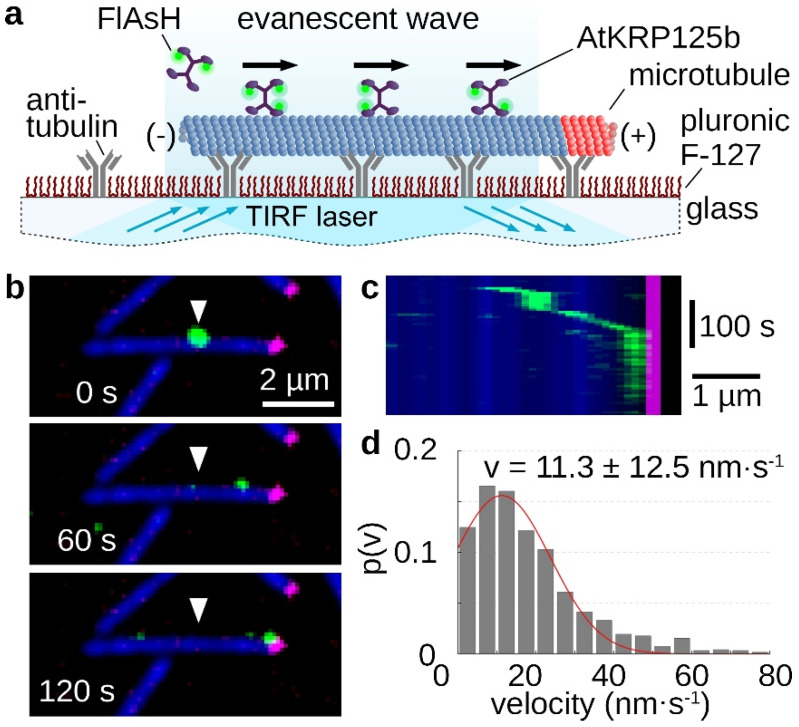
Single molecule stepping assays reveal, AtKRP125b is a processive motor protein. (**a**) Schematic representation of a single molecule stepping assay with fluorescently-labelled full length AtKRP125b. (**b**) Fluorescent micrographs of a single AtKRP125b molecule (green) stepping along an immobilized polarity marked microtubule at different timepoints. The plus end of the polarity-marked microtubule is labelled in magenta, while the remainder of the microtubule is labelled in blue. The starting position of the AtKRP125b-molecule is marked by a white arrowhead. (**c**) Kymograph of the stepping event shown in (**b**). (**d**) Histogram of the mean transport velocities for 121 single molecule stepping events. A Gaussian fit (red line) shows a velocity of 11.3 ± 12.5 nm s^−1^ (mean ± SD).

The processivity of a kinesin describes its ability to move as a single molecule over longer distances along microtubules, always with at least one motor head bound to the microtubule. The longer the traveled distance of this motor protein, the more processive it is. For the role in the mitotic spindle, high processivity is not necessary, because in this case several molecules cooperate. Therefore, kinesin-5 motor proteins can distribute the resulting forces very well among several motor proteins in a cluster and prevent the detachment of individual molecules [20]. In the stepping assay, single FlAsH-stained AtKRP125b molecules moved along microtubules, which were tightly bound to the glass surface via antibodies (Figure 2a). It is clearly evident that AtKRP125b moves processively in the µm range (Figure 2b,c, Supplementary Movie S2). An evaluation of the velocity of the single molecules revealed a mean velocity of 11.3 ± 12.5 nm/s (mean ± SD, n = 122 total stepping events), which is comparable to the velocity from the gliding assay (Figure 2d and Figure 1d).

### 2.3. AtKRP125b Moves along Microtubules toward Their plus End

The directionality of a kinesin is mainly determined by the location of the motor domain and a specific neck-linker sequence [21]. Since the plus ends of spindle microtubules point toward the midzone and kinesin-5 is involved in elongation, i.e., pushing apart the overlapping spindle molecules, AtKRP125b was hypothesized to be a plus end-directed motor protein to fulfill the classical role of a kinesin-5 in the mitotic spindle. In the applied stepping assay, it could be seen that AtKRP125b molecules always moved toward the labeled plus end (Figure 2b,c, Supplementary Movie S2). Accordingly, it could be observed that the displacement of microtubules was unidirectional with a leading minus end and a trailing plus end (labelled end) in the gliding assay (Figure 1b,c and Supplementary Movie S1), indicating that AtKRP125b moved toward the plus end of the microtubule and thus it is a plus end-directed kinesin. This finding is consistent with the directionality determined for human Eg5, which also moves toward the plus end [5].

### 2.4. AtKRP125b Is Able to Cross-Link Microtubules and Slide Them Apart

**Figure 3:**
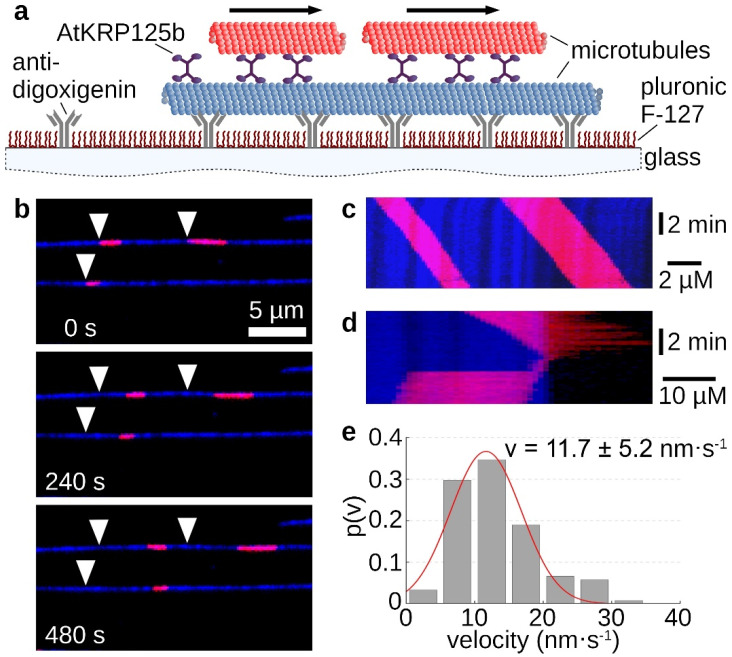
Sliding assays show AtKRP125b cross-links microtubules and slides them apart. (**a**) Schematic representation of a sliding motility assay with full-length AtKRP125b. (**b**) Fluorescent micrographs of short red microtubules sliding along long, immobilized microtubules (blue) at different timepoints. The starting position of each microtubule is marked by a white arrowhead. (**c**) Kymograph of the upper blue microtubule shown in (**b**). (**d**) Kymograph of a short, red microtubule being transported to the end of the lower, blue microtubule and immediately flipping back onto it. The red microtubule stops after flipping. (**e**) Histogram of the mean transport velocities for 122 sliding microtubule filaments. A gaussian fit (red line) shows a velocity of 11.7 ± 5.2 nm s^−1^ (mean ± SD).

The homotetrameric structure of kinesin-5 proteins generally enables two microtubules to be linked and slid apart simultaneously. In the sliding assay, fluorescently labelled long microtubules were tightly bound to the support via antibodies. Free short microtubules labeled with a fluorescent dye of a different wavelength were cross-linked to the immobilized microtubule by recombinant AtKRP125b (Figure 3a). Movement of the short microtubules along the long microtubules occurred only in some cases (Figure 3b,c, Supplementary Movie S3). Moreover, there were rare cases in which the short microtubule reached the end of the long microtubule and, instead of detaching, made a 180° turn and re-attached to the long microtubule. In these cases, the movement stopped (Figure 3d, Supplementary Movie S4). If the plus ends of the microtubules point in the same direction, force is exerted in the same direction by both pairs of motor domains and no net motion occurs. However, if the microtubules are in an anti-parallel relationship, force from the two pairs of motor domains will be exerted in opposite directions and there will be net motion. This motion should be twice as fast as the determined velocity from the gliding assay. Surprisingly, we determined a mean velocity of 11.7 ± 5.2 nm s^−1^ (mean ± SD, N = 121 total sliding events, Figure 3e) for the moving short microtubules, which was not a doubling but even a slight slowdown compared to the gliding velocity of 17 nm s^−1^.

### 2.5. The AtKRP125b Promoter Is Not Active in Mitotically Active Tissues

**Figure 4:**
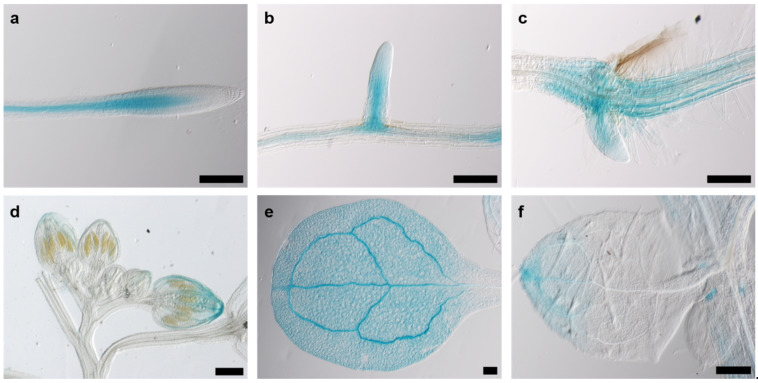
The AtKRP125b promoter is active in various organs. Images show GUS-staining in 8 day-old plants of *pAtKRP125b*::GUS lines after 90 min incubation with X-Gluc. (**a**,**b**), Intense GUS staining was observed in vascular tissue of the root (**a**) and emerging lateral root (**b**) above the meristem. (**c**) GUS staining was visible in the hypocotyl and in emerging adventitious roots. d,e,f, GUS-staining was observed in the apex and vascular tissue of flower buds (**d**), cotyledons (**e**), and true leaves (**f**). Scale bars: 200 µm.

Since cell division in plants occurs almost exclusively in meristematic tissue, we assumed that the promoter activity of *AtKRP125b* is most pronounced in or even restricted to meristems. GUS staining of eight-day-old *pAtKRP125b::GUS* seedlings revealed blue staining of leaves and roots. Promoter activity was observed in vascular tissue in the root (Figure 4a), in emerging lateral roots (Figure 4b), in the basal region of the hypocotyl (Figure 4c), in the petals of young flowers (Figure 4d), and the apex and vascular tissue of cotyledons and true leaves (Figure 4e,f). In 24-day-old *pAtKRP125b::GUS* plants, the promoter was also active in vascular tissue of petals, the stigma, and anthers of mature flowers and in the remnants of the stigma and in the internode of young siliques (data not shown). Remarkably, no GUS staining was detected in mitotically active tissue such as the root apical meristem or the flowering meristem, which contradicts our hypothesis that AtKRP125b shows a role in the mitotic spindle. However, we cannot exclude the possibility that not all regulatory elements were captured with the selected promoter sequence. A repeat of the experiment with a longer promoter sequence failed to transform with agrobacteria due to the length of the construct.

### 2.6. AtKRP125b Knockout Mutants Show No Developmental Abnormalities

If AtKRP125b is indeed involved in mitotic spindle formation, knockout of the corresponding gene should result in changes of plant development, such as growth retardation. However, phenotyping of *krp125b* single mutants did not show any significant change in growth or development compared with wild-type plants (Figure S1). It may, however, well be that one of the other kinesin-5 family members substitutes for the missing *AtKRP125b* function. All our attempts to generate double or multiple kinesin-5 Arabidopsis mutants by CRISPR-Cas9 technology were not successful.

### 2.7. GFP-AtKRP125b Is Localized in the Nucleus and in the Cytoplasm

**Figure 5:**
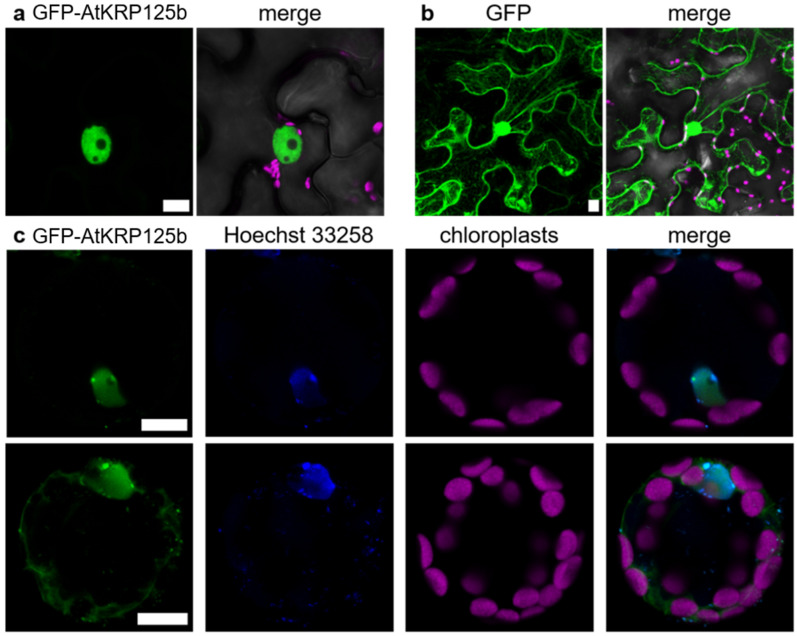
GFP-AtKRP125b is localized in the nucleus and cytoplasm. (**a**) Fluorescent image of transient expressed GFP-AtKRP125b (green) in Nicotiana benthamiana leaf pavement cells. The second image shows a merge of three channels: GFP-AtKRP125b (green), bright field (grey) and chlorophyll autofluorescence (magenta). (**b**) Fluorescent image of transiently expressed free GFP as a comparison to (**a**). Scale bars: 10 µm. (**c**) Co-localization of GFP-AtKRP125b and nucleus in *Arabidopsis thaliana* mesophyll protoplasts. Images show transient expression of GFP-AtKRP125b (green), nuclei staining with Hoechst 33342 (blue) and chloroplast autofluorescence (magenta). The upper images show a protoplast without a signal in the cytoplasm. The lower images show a maximum intensity projection of a protoplast with an additional GFP-AtKRP125b signal in the cytosol.

The lack of *AtKRP125b* promoter activity in mitotically active cells prompted us to test for a putative subcellular localization of the AtKRP125b protein. For that purpose, we infected *Nicotiana benthamiana* leaf pavement cells with *Agrobacteria tumefaciens* carrying a *CaMV-35S::GFP-AtKRP125b* construct and detected the fluorescence by confocal laser scanning microscopy. In contrast to the GFP control, which was distributed between the nucleus and cytoplasm, the GFP-AtKRP125b fusion protein was exclusively found in the nucleus (Figure 5a,b). To quantify the overlap of the signals, we calculated the square of Pearson’s correlation coefficient (R^2^), which was 0.77 and 0.62, respectively, indicating high signal agreement. In the next step, we were aiming to study the subcellular localization in the native environment and transformed isolated *Arabidopsis* leaf cell protoplasts. A strong GFP fluorescence was observed in a nuclear structure. The overlap with the applied Hoechst 33342 signal, which stained DNA, confirmed that the GFP-AtKRP125b protein was indeed localized to the nucleus (Figure 5c). In addition to this expected localization, a fluorescence signal was present in the cytosol in about 50% of all protoplasts (Figure 5c), thus indicating that AtKRP125b may also have a function outside the nucleus during interphase.

## 3. Discussion

Members of the kinesin-5 motor protein subfamily have a major function in the spindle organization during mitosis in different organisms ranging from humans to plants [9]. Here, we demonstrated that the biochemical and biophysical properties of AtKRP125b, one of four kinesin-5 proteins in *Arabidopsis*, were in line with a putative function in spindle organization. Its intracellular localization in nucleus and cytoplasm may suggest an additional function during interphase.

The ability to cross-link the spindle microtubules and thus to maintain the stability of the mitotic spindle depends on the property of kinesin-5 proteins to bind to two different microtubules at the same time and to displace them against each other. The tetrameric nature of kinesin-5 allows the cross-linking of microtubules with its two pairs of motor domains at each end. Though this function has been widely observed in all eukaryotes and is well investigated in animals [5,6,7,11], it had not been demonstrated yet in vitro for a kinesin-5 motor protein from plants. In the first step, we were interested to find out whether AtKRP125b is a plus-end directed motor protein. Studies on the directionality showed that most kinesin-5 proteins, which were localized in the midzone of the mitotic spindle and were responsible for spindle elongation, were plus end-directed motor proteins. An exception is the two yeast kinesins Kip1 and Cin8, which were described as bidirectional kinesins [22,23,24]. In the case of Cin8 bidirectionality is achieved by clustering of multiple molecules. This bidirectionality may indicate a function of Cin8 unrelated to the mitotic spindle [24]. In contrast, kinesin-5 from tobacco TKRP125 shows distinct plus end-directed movement [10,25]. We performed gliding assays and found that AtKRP125 was capable of binding to microtubules and to move towards the plus-end of polarity-marked microtubules (Figure 1a–c and Supplementary Movie S1). We determined the velocity of gliding microtubule filaments to 17 ± 8.8 nm s^−1^ (mean ± SD, n = 504 total transport events, Figure 1d). This relatively slow velocity was comparable to studies on Eg5, showing that Eg5 moves at a velocity of ~20 nm s^−1^ [5]). Such slow velocities are common for kinesins that work collectively in a cluster. These motor proteins are usually non-processive kinesins, because they distribute the resulting forces among several motor proteins in the cluster and prevent the detachment of individual molecules [20]. Although it is not required to be processive to slide microtubules [26,27,28,29], we wanted to know whether AtKRP125b is a processive motor, because such processivity might allow more diverse functions, such as, for example, long range transport. Therefore, we examined the processivity of AtKRP125b in addition to its directionality and velocity. Stepping assays with FlAsH-labelled AtKRP125b revealed a surprisingly high processivity in the µm range (Figure 2b,c). In addition, we have shown in sliding assays that AtKRP125b was indeed able not only to cross-link microtubules but also to move anti-parallel aligned microtubules along each other. Since AtKRP125b exerts force in both directions on microtubules as a bipolar tetramer, one would expect the velocity of transported microtubules to be twice as high as in the gliding assay. In similar in vitro experiments with Eg5, Kapitein et al. [5] observed a doubling of the velocity in sliding assays (~40 nm s^−1^) compared to gliding assays (~20 nm s^−1^) which suits the velocity of microtubules sliding apart at the equator of the metaphase spindle in animals (~30–50 nm s^−1^) [30]. In contrast to that, the velocity in the sliding assay with AtKRP125b was even slightly lower than the velocity in the gliding assay. We cannot rule out the possibility that the low velocity is due to lack of posttranslational modifications in the recombinant molecule, but we rather suspect that the unusually high processivity is associated with the low velocity in the sliding assay. Cryo-EM structure analysis revealed that the tail domain of one kinesin-5 tetramer interacts with the opposing motor domain of a neighboring tetramer [31]. It has been shown that this interaction has a significant impact on the ATP-binding of the motor domain. Thereby, the tail slows down the ATP binding of the motor domain, resulting in a prolonged interaction between the motor domain and microtubule, a slower velocity, and a higher processivity. The reduction of velocity brings an increase of generated force in return (Shimamoto et al. 2015). Since the plant spindle differs in structure from the spindle in animal cells, it is quite possible that this additional force is a prerequisite for AtKRP125b to fulfill its canonical role in the plant cell. In agreement with our data, AtKRP125c was also found to be associated with microtubules and appears to play a central role in the mitotic spindle [11]. Barroso et al. [32] demonstrated binding to microtubules for DcKRP120 from carrot (*Daucus carota*), and Asada et al. [10] demonstrated for TKRP125 from tobacco that this protein moves along microtubules toward their plus end. Both proteins appear to be primarily active during mitosis [10,32]. Furthermore, it is also conceivable that AtKRP125b requires this additional force for other functions unrelated to the mitotic spindle, especially since AtKRP125c is also thought to have a role outside the mitotic spindle [11].

In order to support the notion of AtKRP125b functions in addition to the putative role in the mitotic spindle, we determined the *AtKRP125b* promoter activity and the intracellular localization of the gene product. Since the mitotic spindle is only formed in cells undergoing cell division and these cells are located in mitotic tissue, we assumed to find *AtKRP125b* promoter activity mainly in meristems. In fact, however, GUS staining of *AtKRP125b*-promoter: *GUS* plants showed that the promoter was primarily active in vascular tissue. We did not detect any activity in meristematic tissue (Figure 4). The absence of *AtKRP125b* promoter activity in these regions may contradict our hypothesis that AtKRP125b has a role in the mitotic spindle. However, we cannot exclude the possibility that not all regulatory elements were captured with the selected promoter sequence or that mRNA produced in the vasculature may be transported into mitotically active tissues. Previously, expression analyses indeed showed that the *AtKRP125b* transcript was present in meristematic tissues and induced in mitosis [25]. The high basal level of *AtKRP125b* transcript before the induction of mitosis may suggest that *AtKRP125b* is expressed already in interphase and has a function that is beyond mitosis [25]. This was supported by the detection of GFP-AtKRP125b fusion proteins not only in the nucleus, but also in the cytoplasm (Figure 5). Future experiments will show whether this dual localization is due to shuttling of AtKRP125b between cytoplasm and nucleus or whether AtKRP125b has an additional function during interphase. Such functions outside the mitotic spindle have already been described for animal homologs of kinesin-5. Thus, human Eg5 plays a role in the transport of neurotrophins and neurotransmitter receptors in neurons [33]. Falnikar et al. [34] found that when Eg5 is inhibited in migrating neurons, their velocity is increased, but their movement is less directed. Eg5 transports short microtubules along the axons of migrating neurons. When Eg5 is inhibited, the frequency of transport is reduced [34]. In *Drosophila melanogaster*, kinesin-5 Klp61F transports proteins from the Golgi complex to the cell surface in non-mitotic cells [35]. A similar function for AtKRP125b in non-meristematic tissue would be possible. However, this needs to be investigated by further experiments. Not only in animal cells, but also in plant cells, the localization of kinesin-5 indicates a role outside the mitotic spindle. Inhibition of TKRP125 leads to inhibition of translocation of phragmoplast microtubules in tobacco BY-2 cells [10]. In addition, TKRP125 is present along cortical microtubules during S phase [10]. Based on the ability of AtKRP125b to cross-link and counter-shift microtubules, we hypothesize that AtKRP125b is involved in such processes. Kinesin-4 FRA1 is involved in cell wall assembly and appears to play a role in the organization of cortical microtubule networks [36]. Involvement of kinesin-5 in these processes has not yet been investigated but seems possible to us.

Taken together, our in vitro data showed that on the molecular level AtKRP125b displays the canonical properties of a kinesin-5 motor protein. The localization of AtKRP125b in the nucleus and cytoplasm, as well as the missing promotor activity in meristematic tissue, open the possibility for additional functions beyond the spindle elongation for kinesin-5 proteins in plants.

## 4. Material and Methods

### 4.1. Protein Expression, Purification, and Labelling

For the expression plasmid, *AtKRP125b* cDNA was cloned into the pFastBac1-vector (Invitrogen, Waltham, MA, USA) by using overlap extension PCR cloning [37]. In the cloning procedure, a His-tag and tetracystein-tag were added, respectively. After expression in High Five Cells (BTI-TN-5B1-4), recombinant His-tagged protein was purified via metal affinity chromatography (HisTrap by GE Healthcare, Chalfont St Giles, UK), snap-frozen in liquid nitrogen, and stored at −80 °C or used immediately. Microtubules were purified and polymerized as described before [38,39]. Polarity-marked microtubules were polymerized as described previously [40]. Cy5-labelled tubulin was used to prepare the seeds and Dylight 594-labelled tubulin for the elongation, resulting in Dylight 594-labelled plus ends. Digoxigenin-labelled microtubules were prepared as described previously [39] using a mixture of digoxygenin-labelled tubulin and Cy5-labelled tubulin in a 1:5 ratio.

### 4.2. In Vitro Motility Assays

Microscope flow chambers were constructed as described previously [41]. Gliding and stepping assays were performed as described before [42,43]. AtKRP125b was stained with 1 µM FlAsH-EDT2 [44] for one hour on ice before imaging stepping assays. For the sliding assay, digoxigenin-labelled microtubules were immobilized on the glass surface with digoxigenin antibodies (Roche, Basel, Switzerland). Full-length AtKRP125b was injected into the flow chamber and attached to the digoxygenin-labelled microtubules. Unbound motor protein was washed out after short incubation with BRB80 pH 6.9 containing 0.01 mM Taxol. Then, short Dylight 594-labelled microtubules were injected and washed out after short incubation. All motility assays were performed at 28 °C in BRB80 pH 6.9 supplemented with 1 mM ATP, 10 mM DTT, 10 µM Taxol and 1 mM n-propyl gallate [45]. Fluorescently labelled microtubules were visualized with epi-illumination under a fluorescence microscope (TI-E, Nikon, Tokyo, Japan) at 564 nm or 640 nm, respectively. Single FlAsH-stained AtKRP125b molecules in stepping assays were excited with 488 nm TIRF illumination. Positions of microtubule filaments and single motor protein molecules were tracked with the FIESTA software [46], and velocities were analyzed using MatLab (Mathworks, Natick, MA, USA).

### 4.3. Analysis of Promoter Activity

For cloning of the *AtKRP125b*-promoter GUS construct we amplified 847 bp in front of the start codon of *AtKRP125b* from genomic DNA by PCR with primers Kin5_GUS-fw (CACCGTGTTTCCTAGCCTTCTTTT) and Kin5_GUS-rev (GAACGGACGGCGAATCCAGTGAGA) and cloned the derived fragment into pMDC163 using TOPO PCR-Cloning (Invitrogen, Waltham, MA, USA). *Agrobacteria tumefaciens* C58C1 cells were transformed with this plasmid and painted onto flowers and buds of *Arabidopsis thaliana* plants as previously described [47]. Transformed offspring was identified by hygromycin selection [48]. We identified five independent GUS-positive plant lines that showed a distinct signal after staining in X-Gluc solution for 90 min.

### 4.4. Plant Material and Growth Conditions

If not stated otherwise, all plants were grown either on soil or Murashige and Skoog media (MS, Duchefa, Haarlem, The Netherlands) supplemented with 0.8% phyto agar (Duchefa) and 1% sucrose in growth chambers at 22 °C under long-day conditions (16 h light, 8 h dark). To examine *Arabidopsis thaliana* roots, seedlings were grown at 22 °C and 17 °C in a vertical position for seven days under long-day conditions (16 h light, 8 h dark) and afterwards shifted to new plates to ensure that the roots grew on top of MS media. The roots were exposed to light during growth on MS medium. 72 plants of each line were examined. The T-DNA alleles of *atkrp125b-1* (GK011.H10) and *atkrp125b-2* (SALK_152139) were received from the Nottingham Arabidopsis Stock Centre.

### 4.5. Subcellular Localization

A GFP-fusion construct was generated by cloning *AtKRP125b* from cDNA into pMDC43 using TOPO-PCR cloning (Invitrogen). Protoplasts from *Arabidopsis thaliana* mesophyll cells were prepared as previously described [49] and transformed, using 20 ng of plasmid DNA. Additionally, the construct was inserted into *A. tumefaciens* C58C1 and then transformed into *N. benthamiana* leaves [50]. These procedures were repeated 3 times. All imaging of subcellular localization was captured with a confocal laser scanning microscope (TCS SP8, Leica, Wetzlar, Germany). GFP fluorescence was visualized using an argon laser at 488 nm for excitation and an emission window of 502–512 nm. Hoechst 33342 was visualized with an excitation of wavelength of 405 nm and an emission window of 445–465 nm, whereas chlorophyll autofluorescence was detected in an emission window of 660–675 nm.

## Supplementary Materials

The following are available online at https://www.mdpi.com/article/10.3390/ijms222111361/s1.
