# Supplementary material for: The Arabidopsis thaliana Kinesin-5 AtKRP125b Is a Processive, Microtubule-Sliding Motor Protein with Putative Plant-Specific Functions
Source: Int J Mol Sci. 2021 Oct 21;22(21):11361. doi: 10.3390/ijms222111361 (PMC8583919; doi:10.3390/ijms222111361)
Supplement: Supplementary file 1 [file ijms-22-11361-s001.zip › supplement.pdf]

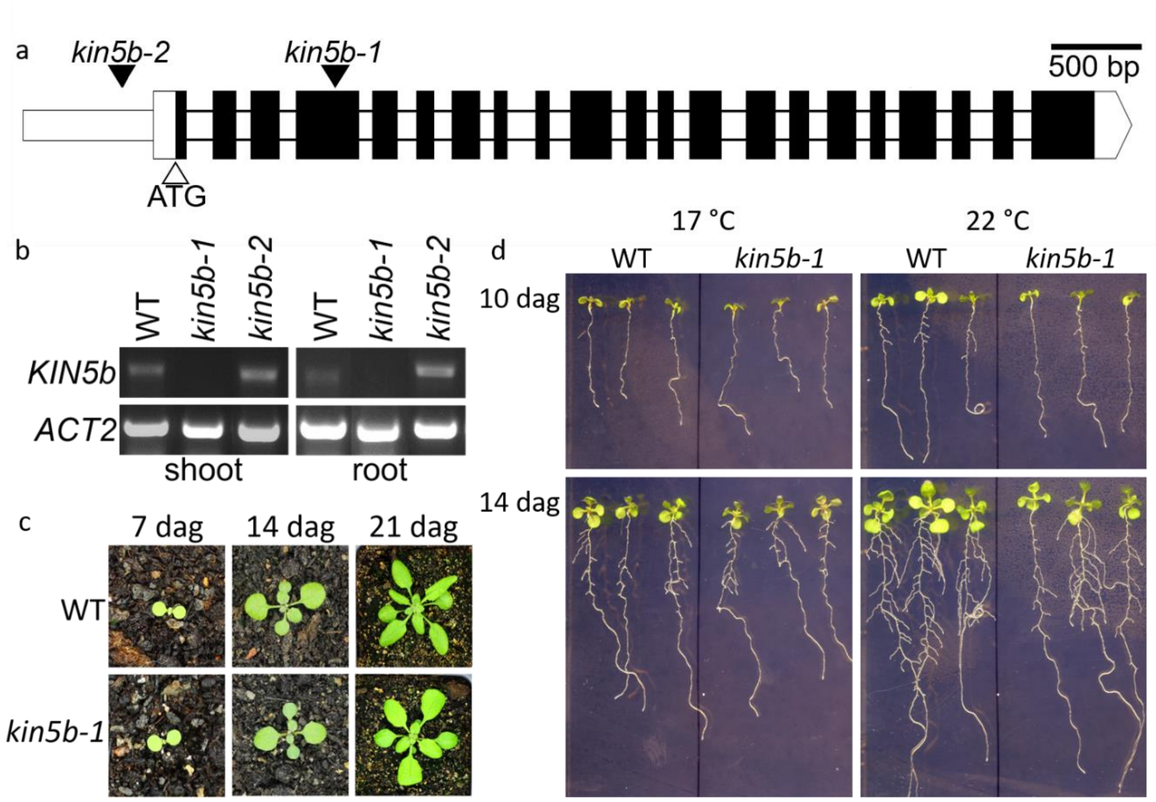

Figure S1: **AtKRP125b knockout mutant shows no phenotype.** a; Exon-intron structure of *At2g36200*/*AtKRP125b*. T-DNA insertion is located in the fourth exon for kin5b-1 and in the intergenomic region for kin5b-2, respectively. b; RT-PCR of kin5b-1 and kin5b-2 of shoot and root material of seven-day old seedlings. kin5b-1 shows no amplification either in shoot nor in root material. A band for kin5b-2, comparable to WT material proves kin5b-2 is no knockout for AtKRP125b. c; Comparison of WT and kin5b-1 plants grown over several weeks under long day conditions at 22 °C show no difference between both lines. d; Comparison of root growth of WT and kin5b-1 plants grown at long day conditions on MS-media at 17 °C and 22 °C. There is no visible difference between both either 10 days after germination (dag), nor 14 dag.

movie 1: **AtKRP125b moves with a slow velocity in in vitro gliding assays**. Polarity-marked microtubules gliding along a AtKRP125b coated surface. The plus end of the polarity-marked microtubule is labelled in red, the remainder of the microtubule is labelled in blue.

movie 2: **Single molecule stepping assays reveal, AtKRP125b is a processive motor protein**. A single AtKRP125b molecule (green) stepping along a immobilized polarity marked microtubule. The plus end of the polarity-marked microtubule is labelled in magenta, while the remainder of the microtubule is labelled in blue

movie 3: **Sliding assays show AtKRP125b crosslinks microtubules and slides them apart.** Short red microtubules sliding along long, immobilized microtubules (blue). The starting position of each microtubule is marked by a white arrowhead.

movie 4*:* **Polarity-dependence of microtubule sliding by AtKRP125b.** A short, red microtubule being transported to the end of the lower, blue micotubule and immedeatly flipping back onto it, thereby reverting the polarity in respect to the previous transport direction. The red microtubule stops after flipping.
