# Supplementary figures and images for: The Arabidopsis thaliana Kinesin-5 AtKRP125b Is a Processive, Microtubule-Sliding Motor Protein with Putative Plant-Specific Functions
Source: Int J Mol Sci. 2021 Oct 21;22(21):11361. doi: 10.3390/ijms222111361 (PMC8583919; doi:10.3390/ijms222111361)

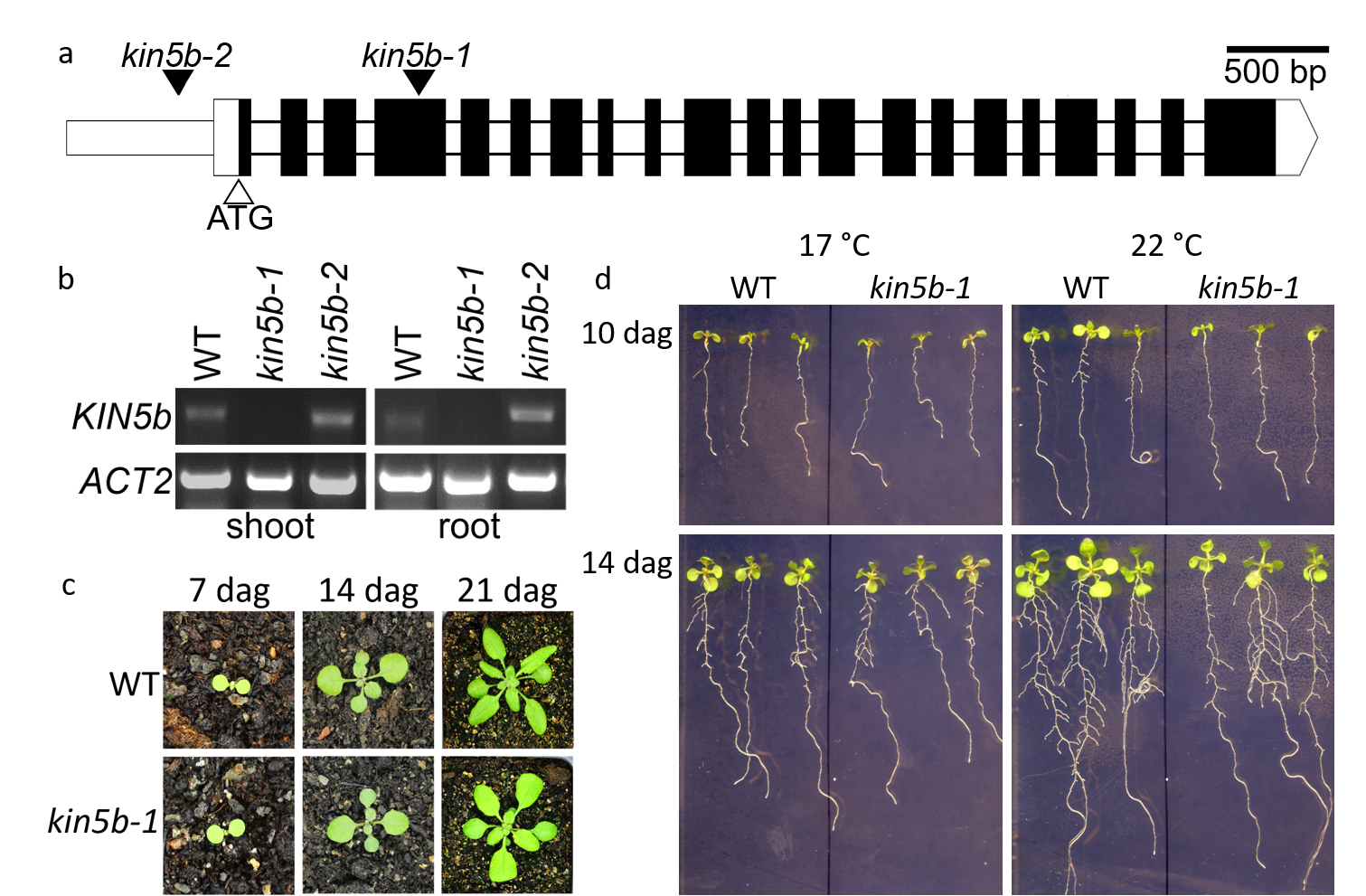

Supplement: Supplementary file 1 [file ijms-22-11361-s001.zip › Supplemental Figure.png]
